# Supplementary material for: Extreme atmospheric rivers in a warming climate
Source: Nat Commun. 2023 Jun 3;14:3219. doi: 10.1038/s41467-023-38980-x (PMC10239457; doi:10.1038/s41467-023-38980-x)
Supplement: Supplementary file 1 — Supplementary Information [file 41467_2023_38980_MOESM1_ESM.pdf]

**Supplementary Information For**

# Extreme Atmospheric Rivers in a Warming Climate

**Shuyu Wang[1], Xiaohui Ma[1,2*], Shenghui Zhou[2], Lixin Wu[1,2], Hong Wang[1,2], Zhili Tang[1], Guangzhi Xu[3] , Zhao Jing[1,2], Zhaohui Chen[1,2], Bolan Gan[1,2]**

1. Frontiers Science Center for Deep Ocean Multispheres and Earth System and Key Laboratory of Physical Oceanography, Ocean University of China, Qingdao, China.
2. Laoshan Laboratory, Qingdao, China.
3. College of Global Change and Earth System Science, Beijing Normal University, Beijing, China

[*]Corresponding author. E-mail: maxiaohui@ouc.edu.cn

**This PDF file includes:**

Supplementary Figs S1-S7

Table S1

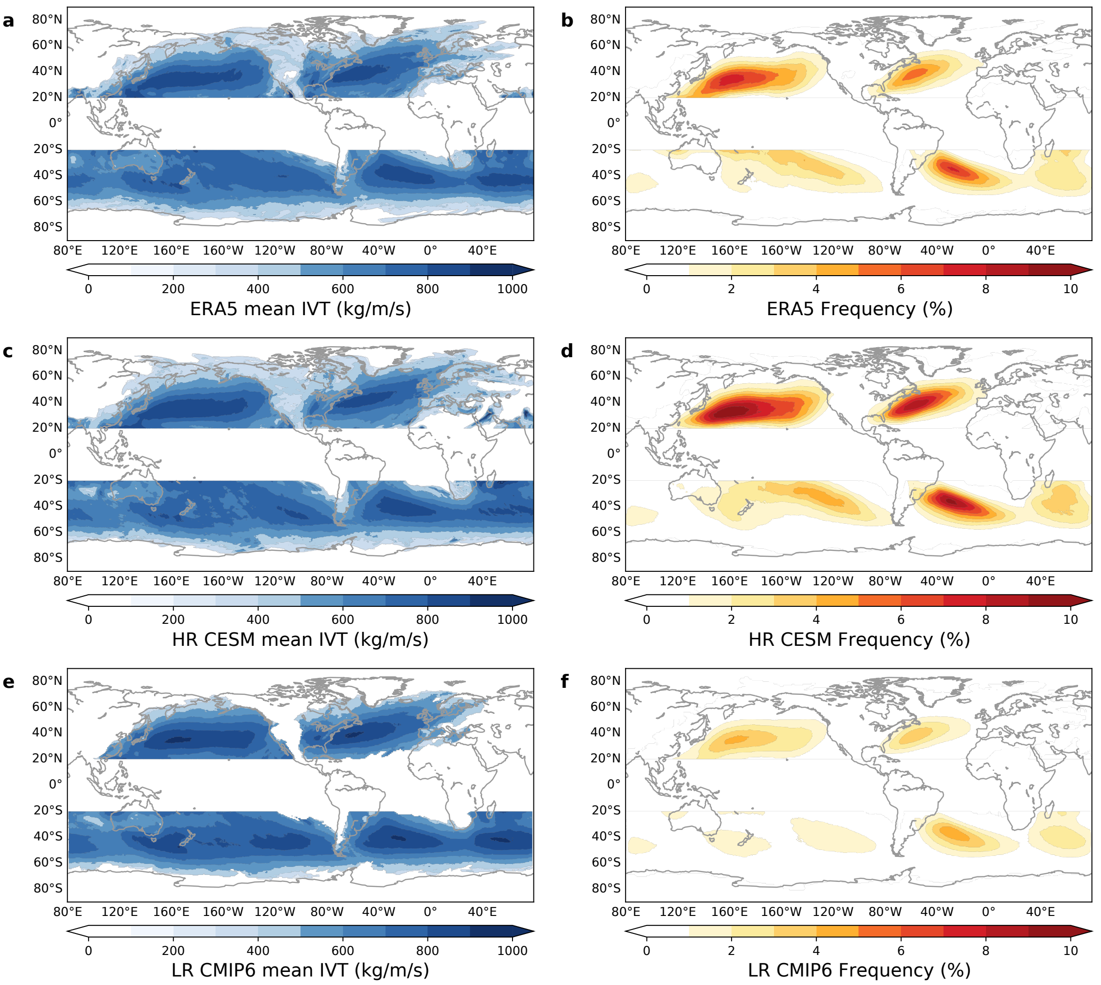


**Figure S1 | Observed and simulated Extreme Atmospheric River (EAR) intensity and occurrence frequency.** The mean intensity of EAR integrated water vapor transport (IVT, kg/m/s) in boreal winter season in ERA5 reanalysis (**a**, the fifth generation European Centre for Medium-Range Weather Forecasts atmospheric reanalysis), HR-CESM (**c**, High Resolution Community Earth System Model) and LR-CMIP6 (**e**, Low Resolution Coupled Model Intercomparison Project Phase 6) during 1979-2005. **b, d**, **f**, as for **a**, **c, e**, but for the mean occurrence frequency of EARs (%). Source data are provided as a Source Data file.

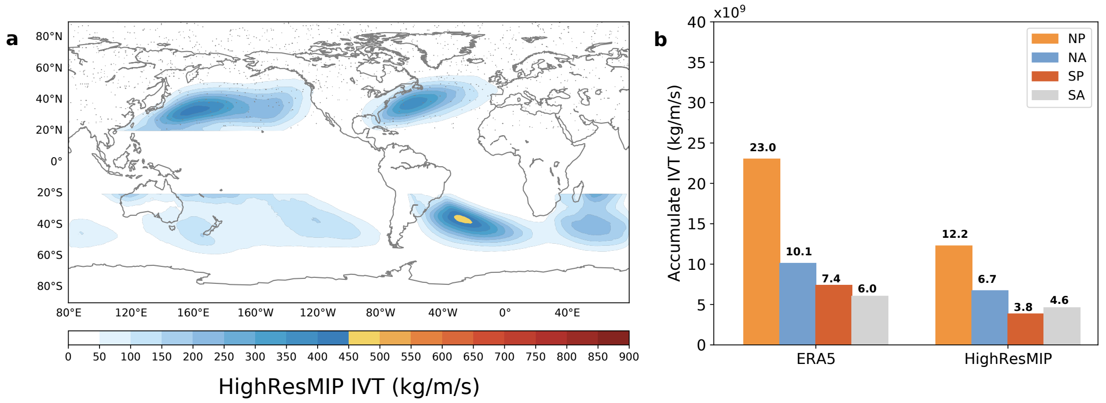


**Figure S2 | Simulated Extreme Atmospheric Rivers (EARs) in High Resolution Model Intercomparison Project (HighResMIP).** Normalized accumulated EAR integrated water vapor transport (IVT, kg/m/s) in boreal winter season (ONDJFM) in HighResMIP during 1979-2005 (**a**). EAR IVT (kg/m/s) averaged in AR-active regions (red boxes outlined in Fig. 2d) in HighResMIP (**b**). Source data are provided as a Source Data file.

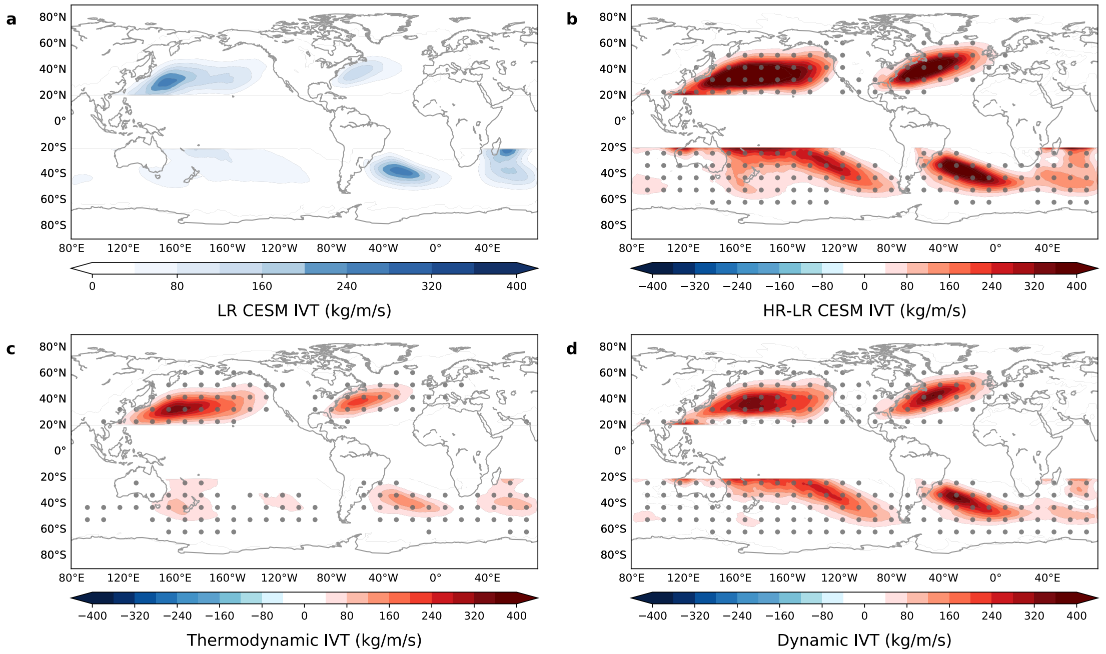


**Figure S3 | Comparison of simulated Extreme Atmospheric Rivers (EARs) between High Resolution (HR) and Low Resolution (LR) Community Earth System Model (CESM).** Normalized accumulated EAR integrated water vapor transport (IVT, kg/m/s) in historical simulations (1956-2005) in LR CESM (**a**) and the difference of that between HR-CESM and LR-CESM (**b**). The thermodynamic (**c**) and dynamic (**d**) changes of EAR IVT (kg/m/s) between HR-CESM and LR-CESM. Source data are provided as a Source Data file.

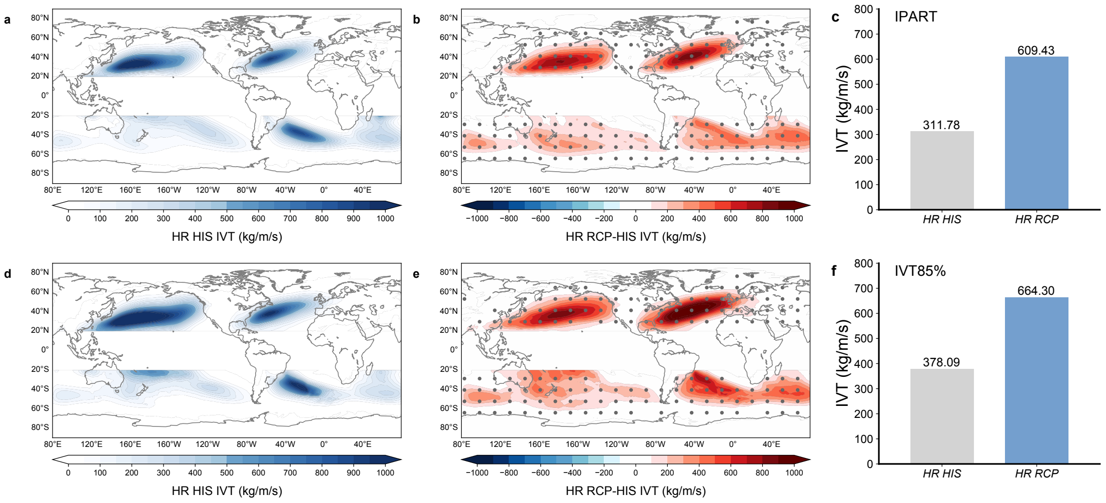


**Figure S4 | Sensitivity of Extreme Atmospheric River (EAR) projections to AR Detection Tools (ARDTs) in High Resolution Community Earth System Model (HR CESM).** Normalized accumulated integrated water vapor transport (**a,** IVT, kg/m/s) simulated in historical simulations (HR-HIS, 1956-2005) and the difference of that between future simulations (HR-RCP, 2051-2100) and HR-HIS (**b**) based on IPART method. (**c**) Global averaged EAR IVT (kg/m/s) in HR-HIS and HR-RCP based on IPART method. **d-f,** as for **a-c**, but for IVT85% ARDT. Source data are provided as a Source Data file.

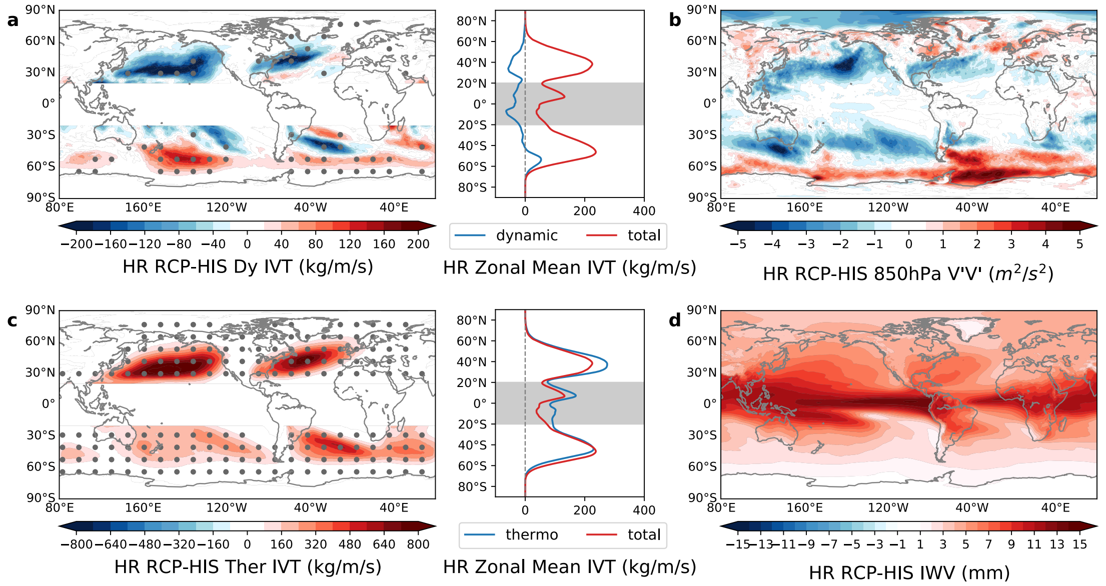


**Figure S5 | Thermodynamic and dynamic Extreme Atmospheric River (EAR) responses in High Resolution Community Earth System Model (HR CESM).** The dynamic (**a**) and thermodynamic (**c**) EAR integrated water vapor transport (IVT) difference between historical (HR-HIS) and future (HR-RCP) simulations and the corresponding zonal averaged value (blue) compared with that of total IVT change (red). Values within the tropics ([20°S-20°N]) are blocked. Note that the sum of dynamic and thermodynamic EAR IVT gives the total IVT change shown in **Fig. 2e** and the color scales in **a&c** are different. The storm track (**b,** 850 hPa $V'V'$, $m^2/s^2$) and integrated water vapor (**d,** IWV, mm) differences between HR-RCP and HR-HIS. Source data are provided as a Source Data file.

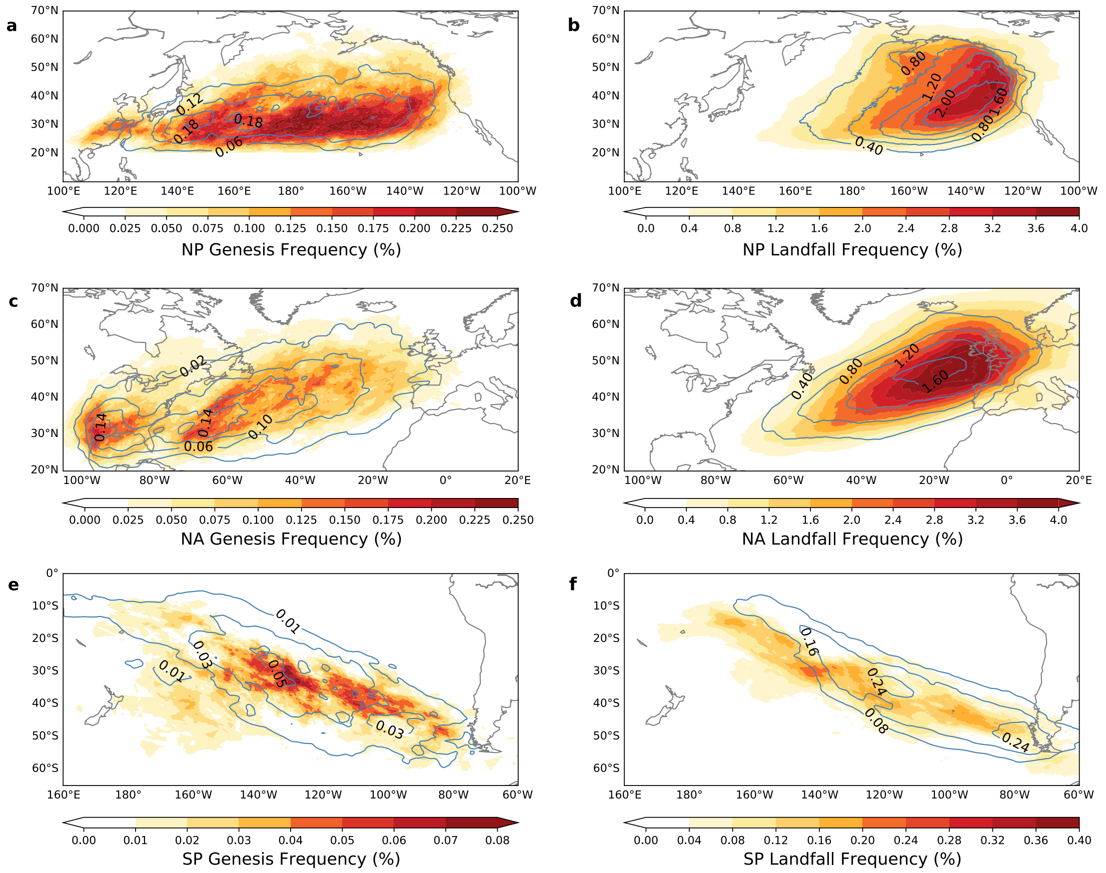


**Figure S6 | Landfalling Extreme Atmospheric River (EAR) responses at different stages in High Resolution Community Earth System Model (HR CESM).** Occurrence frequency (%, a) of landfalling EARs during the genesis stage in historical simulations (HR-HIS, contours) and the difference of that between future simulations (HR-RCP) and HR-HIS (shading) in the North Pacific (NP, **a**), North Atlantic (NA, **c**) and South Pacific (SP, **e**). **b, d**, **f**, as for **a**, **c, e**, but for the occurrence frequency during the landfalling stage (%). Source data are provided as a Source Data file.

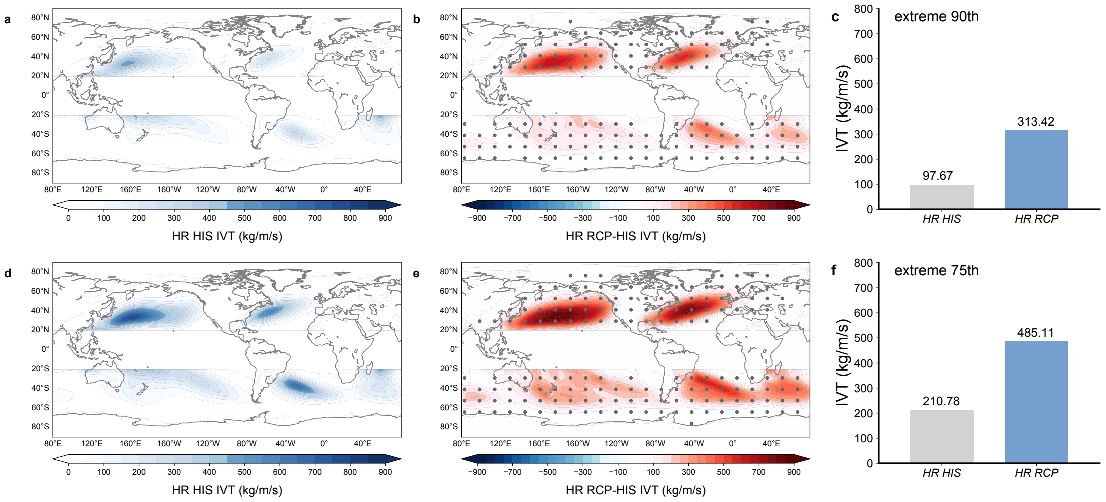


**Figure S7 | Sensitivity of Extreme Atmospheric River (EAR) projections to integrated water vapor transport (IVT) thresholds used to define EARs in High Resolution Community Earth System Model (HR CESM).** Normalized accumulated integrated water vapor transport (**a,** IVT, kg/m/s) simulated in historical simulations (HR-HIS, 1956-2005) and the difference of that between future simulations (HR-RCP, 2051-2100) and HR-HIS (**b**) for EARs defined by the 90th percentile IVT threshold. (**c**) Global averaged EAR IVT (kg/m/s) in HR-HIS and HR-RCP for EARs defined by the 90th percentile IVT threshold. **d-f,** as for **a-c**, but for EARs defined by the 75th percentile IVT threshold. Source data are provided as a Source Data file.

**Table S1 | The percentage of Extreme Atmospheric Rivers (EARs) pairing with Extratropical Cyclones (ECs) in High Resolution Community Earth System Model (HR CESM).** The percentage of EARs and ECs in the North Pacific (NP), North Atlantic (NA) and South Pacific (SP) simulated in historical (HR-HIS, 1956-2005) and the future (HR-RCP, 2051-2100) simulations. Source data are provided as a Source Data file.

| | **NP** | **NA** | **SH** |
|---|---|---|---|
| **HR-HIS** | 84.8% | 83.1% | 82.7% |
| **HR-RCP** | 82.7% | 82.2% | 77.5% |
